# Clinicopathological findings of an MM2-cortical-type sporadic Creutzfeldt-Jakob disease patient with cortical blindness during a course of glaucoma and age-related macular degeneration

**DOI:** 10.1080/19336896.2019.1631680

**Published:** 2019-06-20

**Authors:** Yuichi Hayashi, Yasushi Iwasaki, Masahiro Waza, Hideaki Shibata, Akio Akagi, Akio Kimura, Takashi Inuzuka, Katsuya Satoh, Tetsuyuki Kitamoto, Mari Yoshida, Takayoshi Shimohata

**Affiliations:** a Department of Neurology, Gifu University Graduate School of Medicine, Gifu, Japan; b Autopsy Center of Prion Disease, Institute for Medical Sciences of Aging, Aichi Medical University, Nagakute, Japan; c Department of Neurology, Kakamigahara Rehabilitation Hospital, Kakamigahara, Japan; d Department of Neurology, Gifu Municipal Hospital, Gifu, Japan; e Department of Locomotive Rehabilitation Sciences, Nagasaki University Graduate School of Medicine, Nagasaki, Japan; f Division of CJD Science and Technology, Department of Prion Research, Center for Translational and Advanced Animal Research on Human Diseases, Tohoku University School of Medicine, Sendai, Japan

**Keywords:** Creutzfeldt-Jakob disease, MM2-cortical-type sporadic Creutzfeldt-Jakob disease, cortical blindness, diffusion-weighted MRI, elderly patient, ocular disease, dementia

## Abstract

Here, we report an autopsy-verified patient with MM2-coritical-type sporadic Creutzfeldt-Jakob disease (MM2C-type sCJD) presenting cortical blindness during a course of glaucoma and age-related macular degeneration, and focus on the difficulties involved in early clinical diagnosis. An 83-year-old man was admitted to our hospital 15 months after the onset of cortical blindness, and 9 months after the onset of progressive dementia. Neurological examination revealed dementia, frontal signs, visual disturbance, dysphagia, myoclonus and exaggerated tendon reflexes in the four extremities. Diffusion-weighted MRI (DW-MRI) showed cortical hyperintensities predominantly in the bilateral occipital lobes. *PRNP* gene analysis showed no mutations with methionine homozygosity at codon 129. Cerebrospinal fluid (CSF) examination revealed elevation of 14–3-3 and total tau protein. The symptoms progressed gradually, and the patient died of aspiration pneumonia, 30 months after the onset. Neuropathological examination revealed extensive large confluent vacuole-type spongiform changes in the cerebral cortices. Prion protein (PrP) immunostaining showed perivascular and plaque-type PrP deposits. We diagnosed our patient as MM2C-type sCJD. There are two difficulties in the early clinical diagnosis of MM2C-type sCJD with ocular disease in the elderly; delayed utilization of DW-MRI, and accompaniment of ocular disease. For early diagnosis of MM2C-type sCJD, we conclude that clinician should perform DW-MRI for patients with isolated dementia or cortical visual disturbance.

## Introduction

MM2-cortical-type sporadic Creutzfeldt-Jakob disease (MM2C-type sCJD) develops as fatal, progressive dementia with relatively slow progression. The frequency of MM2C-type sCJD among sCJD patients have been reported to be 2% of sCJD and 6.7% in the Caucasian and the Japanese population, respectively [1]. Previously, we reported a probable MM2C-type sCJD patient whose clinical course involved presentations resembling the Heidenhain variant form of CJD, with the onset of isolated visual disturbance, and later development of dementia [2]. Early clinical symptoms of MM2C-type sCJD do not always satisfy the revised World Health Organization (WHO) diagnostic criteria for sCJD. Some patients, in the early stages of MM2C-type sCJD, may be misdiagnosed with Alzheimer’s disease (AD) [3] or ocular disease. Additionally, elderly patients frequently accompanied with ocular diseases. Therefore, diagnosis of visual symptoms due to CJD pathology may be delayed.

Here, we report a patient with autopsy-verified MM2C-type sCJD presenting cortical blindness during a course of glaucoma and age-related macular degeneration, and focus on the difficulties associated with the early clinical diagnosis of MM2C-type sCJD.

## Patient and methods

### Clinical summary

An 83-year-old man with a prior, 2-year history of glaucoma and age-related macular degeneration of the right and left eyes, respectively, initially complained of rapidly progressive cortical blindness in February 2016. He was admitted to the ophthalmology department; however, a diagnosis could not be made. Moreover, he complained of cognitive impairment and disorientation in August 2016, and these symptoms deteriorated further. He was admitted with a home care doctor, and was diagnosed with AD; however, an MRI was not performed. He presented with 15-month and 9-month histories of cortical blindness and progressive dementia, respectively, before admission to our department. Neurological examination revealed dementia, frontal signs, visual disturbance, dysphagia, myoclonus and exaggerated tendon reflexes in the four extremities. He exhibited state of almost akinetic mutism. Cerebrospinal fluid (CSF) examination [4] revealed normal nuclear cells with total protein (68 mg/dL) and, elevation of 14–3-3 (>500 μg/mL) and total tau (>2,200 pg/mL) proteins. Prion proteins (PrP) in the CSF were amplified by the real-time quaking-induced conversion method as described previously [5].

**Figure 1.**
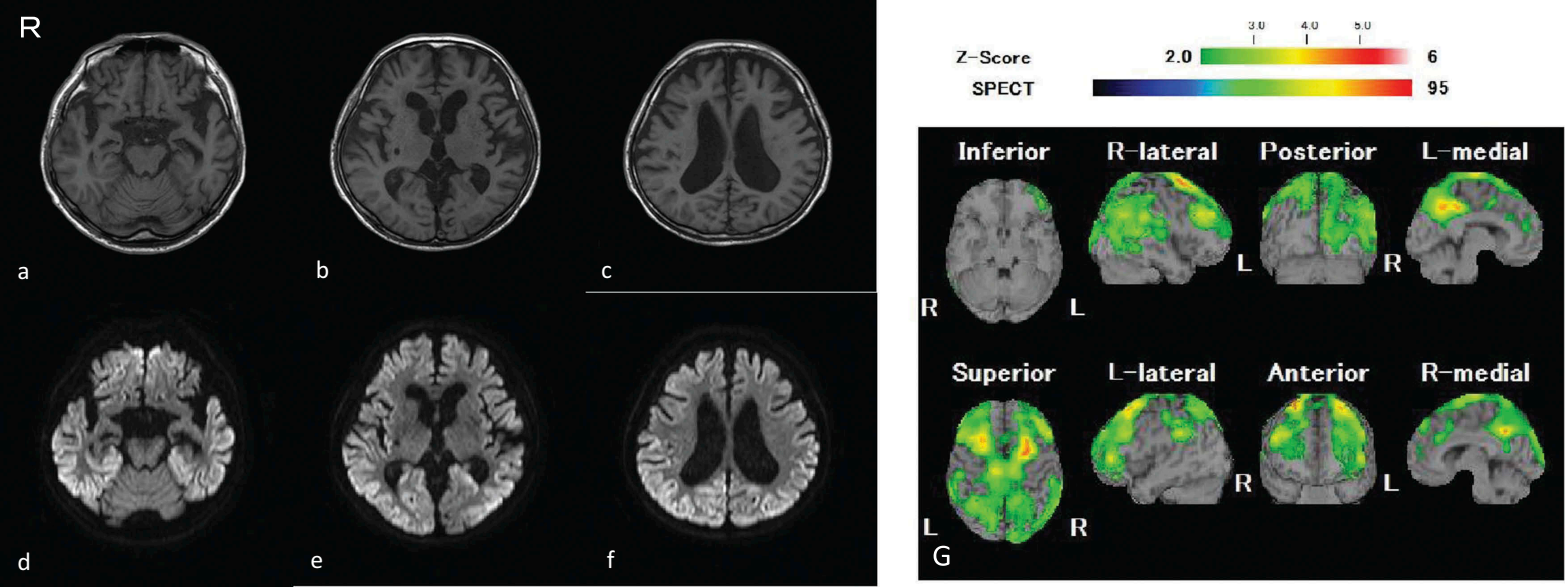
MR images and SPECT obtained 15 months after the onset of symptom. T1-weghted MR images (a-c); diffusion-weighted images (d-f), an easy Z-score (eZIS) analysis of ^9m^Tc-etyhlcysteinate dimer-single photon emission computed tomography (^99m^Tc-ECD-SPECT) image (g). The Z-score scale of 2.0 to 6 is indicated by a green to red (lower regional cerebral blood flow (rCBF)) color gradient in panel g. R: right side; L: left side. T1-weighted MRI showed bilateral frontotemporal atrophy and symmetric enlarged lateral ventricles predominantly posterior horn due to occipital atrophy (a-c). Diffusion-weighted MRI showed cortical hyper-intensities in the bilateral frontal, temporal parietal and occipital cortices without obvious signal changes in the basal ganglia 15 months after the initial symptom (d-f). An eZIS analysis of ^99m^Tc-ECD-SPECT showed decreased rCBF in the bilateral frontal, parietal, and right posterior cortices 15 months after the onset (g).

T1-weighted MRI showed bilateral frontotemporal atrophy and symmetrically enlarged lateral ventricles of the posterior horn due to occipital atrophy (Figure 1a-c). 1.5-Tesla diffusion-weighted MRI (DW-MRI) showed cortical hyper-intensities in the bilateral frontal, temporal parietal and occipital lobes without obvious signal changes in the basal ganglia 15 months after the initial symptom (Figure 1d-f). An easy Z-score (eZIS) analysis (Fujifilm RI Pharma, Tokyo, Japan) of ^99m^Tc-etyhlcysteinate dimer-single photon emission computed tomography (^99m^Tc-ECD-SPECT) showed decreased regional cerebral blood flow (rCBF) in the bilateral frontal, parietal, and right posterior cortices, 15 months after the onset (Figure 1g). Serial electroencephalogram revealed no periodic sharp wave complexes during the disease course of the disease. *PRNP* gene analysis showed no mutations with methionine homozygosity at codon 129. The patient’s slowly progressing course of the disease and MRI findings strongly suggested a clinical diagnosis of MM2C-type sCJD.

He received percutaneous endoscopic gastrostomy in June 2017, and he was transferred to a chronic care hospital. He was followed-up by our medical network for prion disease [6]. His symptoms gradually progressed, and he died of aspiration pneumonia 30 months after the onset. We had obtained an informed consent for autopsy from his family, and transferred the patient’s body to the Autopsy Center of Prion Disease.

### Neuropathological examination

A postmortem study was performed 18h after death. The brain was fixed in 20% neutral-buffered formalin for 4 weeks, and tissue blocks were immersed in 95% formic acid for 1h to inactive prion infectivity. Subsequently, the specimens were embedded in paraffin and cut into 4-μm sections. The sections were deparaffinized in lemosol, rehydrated through an ethanol gradient, and stained. For routine neuropathological examinations, sections were stained with hematoxylin-eosin, Klüver-Barrera’s, and modified Gallyas-Braak silver stains.

Immunohistochemical analysis was performed using monoclonal antibody against PrP (3F4; Dako, Glostrup, Denmark, mouse monoclonal, diluted 1:100) after hydrolytic autoclaving for antigen retrieval [7]. PrP immunostaining was conducted as previously described [8]. Immunostaining with anti-Aβ (4G8; Signet. Dedham, MA, mouse monoclonal, diluted 1:2,000) and anti-hyperphosphorylated tau (AT-8; Innogenetics, Ghent, Belgium, mouse monoclonal, diluted 1:1,000), and anti-phosphorylated α-synuclein (pSyn#64; Wako Pure Chemical Industries, Osaka, Japan, mouse monoclonal, 1:3,000) was also performed. In these immunostainings, the binding of the primary antibody was detected using the envision amplified visualization method (En Vision plus kit; Dako). Peroxidase-conjugated streptavidin was visualized using 3, 3ʹ-diaminobenzidine (DAB; Wako Pure Chemical Industries) as the final chromogen. The immunostained sections were lightly counterstained with Mayer’s hematoxylin.

### Western blot analysis of protease-resistant PrP

The cryopreserved right frontal cerebral cortex, which was snap frozen and stored at −80°C prior to use, was homogenized, and Western blot analysis of protease-resistant PrP (PrP^sc^) was performed using 3F4 antibody as previous described [8]. PrP^sc^ typing was performed according to the sCJD classification system proposed by Parchi, *et al* [9]. Furthermore, to investigate the co-occurrence of type 1 and type 2 PrP^sc^, we carried out an additional experiment using the type 1 PrP^sc^-specific (Tohoku-1) and type 2 PrP^sc^-specific (Tohoku-2) antibodies after protease treatment [10].

## Results

### Macroscopic findings

The patient’s post-mortem brain weight was 1,340g. Mild cerebral atrophy was found on macroscopic analysis; however, no apparent atrophy was observed in the basal ganglia, brainstem, and cerebellum.

### Microscopic findings

**Figure 2.**
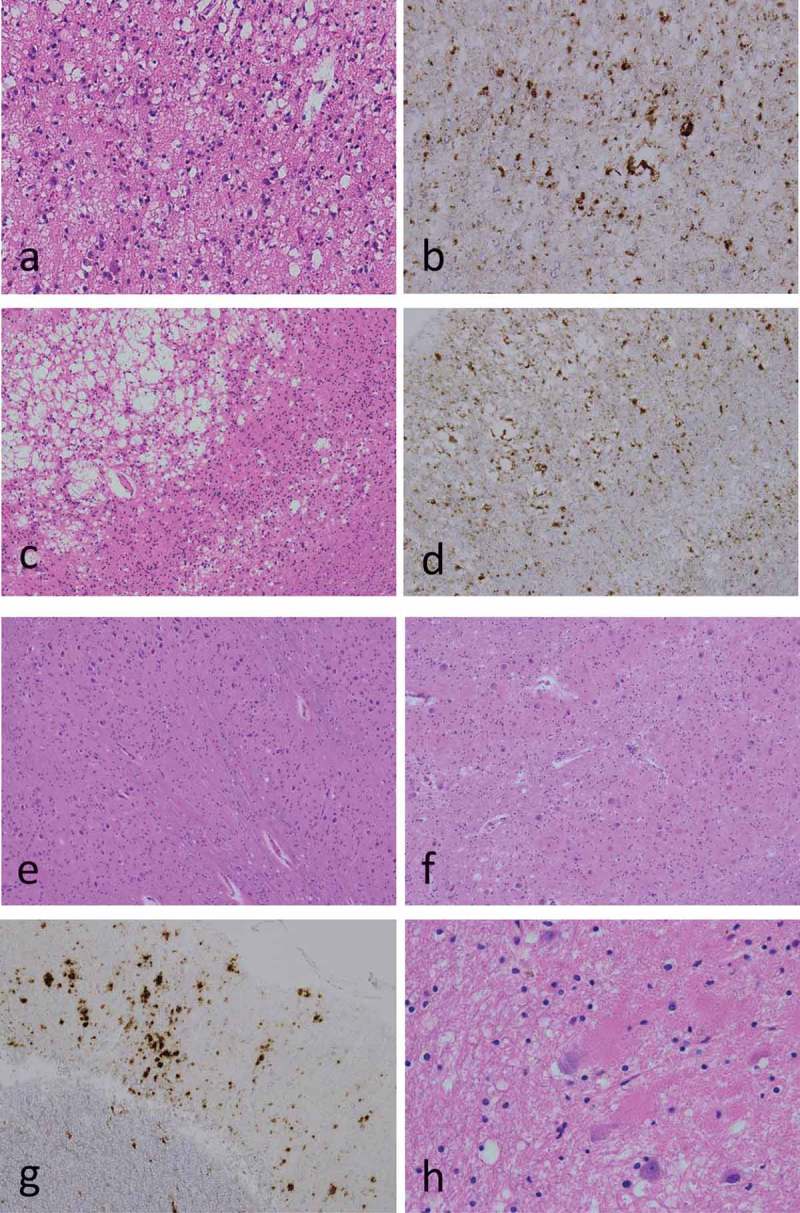
Microscopic findings. Hematoxylin-eosin stains (a, c, e, f, h); anti-PrP immunostains using 3F4 antibodies (b, d, g); middle temporal gyrus (a, b); primary visual cortex (striate area) (c, d); inferior olivary nuclei (e); cerebellar cortex (f, g); cerebellar dentate nuclei (h). Neuropathological examination revealed large confluent vacuole type spongiform changes in the neocortices (a, c); however, no spongiform changes were found in the inferior olivary nuclei (e). PrP immunostaining showed PrP deposits around the vacuoles (perivacuole-type), and rough plaque-type PrP deposits (coarse-type) in mainly in the neocortices (b, d, g). Grumose degeneration was found in the cerebellar dentate nuclei (h).

Neuropathological examination revealed large confluent vacuole type spongiform changes in the cerebral neocortices (Figure 2a, 2c); however, no spongiform changes were found in the inferior olivary nuclei (Figure 2e). Moreover, relatively mild neuronal loss and hypertrophic astrocytes were noted. Mild neurodegeneration was observed in the basal ganglia, especially the striatum, and the thalamus. Interestingly, grumose degeneration was found in the cerebellar dentate nucleus (Figure 2h).

### Prp immunohistochemical findings

Immunostaining for PrP showed prominent perivacuolar-type, and rough plaque-type (coarse-type) PrP deposits mainly in the cerebral neocortices (Figure 2b, 2d), and mild in the basal ganglia, the thalamus, and the cerebellar cortices (Figure 2g). The brainstem, including inferior olive nuclei, exhibited almost no immunostaining for PrP.

### Aging pathology

Aging pathology was mild. Braak neurofibrillary tangles (NFT) stage III or AT8 NFT stage III were found in the parahippocampus and the entorhinal cortex. AD pathology was very mild with no presence of senile plaque or amyloid angiopathic change. Lewy body, or argyrophilic grain were not observed. TDP-43 or α-synuclein immunopositive staining was not observed. Slight microinfarctions were noted; however, the sclerotic changes were apparently in the main arteries and arterioles of the brain.

## Western blot analysis of protease-resistant PrP

**Figure 3.**
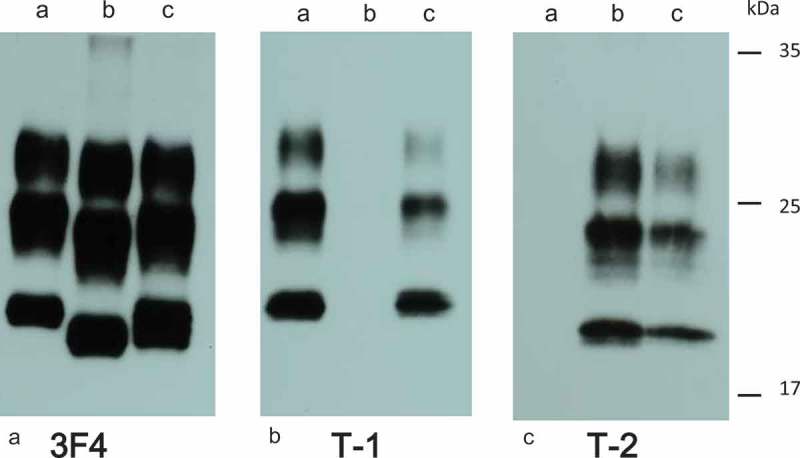
Western blot analysis of protease-resistant PrP. Western blot analysis using 3F4 antibodies (a), Tohoku-1 (b), or Tohoku-2 (c). Brain homogenate sample from MM1 patient (a), the current patient (b), or MM1 + 2 patient (c). Panels showed type 2 PrP^sc^, with 19 kDa unglycosylated band in the current patient.

Western blot analysis using 3F4 antibody revealed the presence of type 2 PrP^sc^, as 19 kDa unglycosylated band (Figure 3a). Moreover, Western blot analyzes using type 2 PrP^sc^-specific antibody showed the presence of apparent positive bands, whereas, no positive bands were observed with type 1 PrP^sc^-specific antibody (Figure 3b, 3c). According to the sCJD classification system [9], the patient was classified as MM2C-type sCJD.

### Summary of pathological findings

The results of pathological examination showed positive confluent vacuoles and coarse/perivacuolar deposits in the cerebral cortices with negative synaptic deposits, at least in the cerebellum. Moreover, pathological changes in the inferior olivary nuclei were extremely mild. According to the consensus classification of human prion disease histotypes [11], we classified the present patient as a pure MM2C-type sCJD.

Interestingly, grumose degeneration was found in the cerebellar dentate nucleus. Grumose degeneration, whose significance remains unknown, is generally observed in progressive supranuclear palsy [12], and other neurodegenerative diseases such as Machado-Joseph disease, corticobasal degeneration (CBD) and Huntington disease; however, the current patient showed no such pathological accompaniments.

## Discussion

This case report focuses on the difficulties associated with the early clinical diagnosis of MM2C-type sCJD with ocular disease in elderly patients. The current patient had been diagnosed with AD until a DW-MRI was performed. Since no diagnosis of CJD was made until 15 months after symptom onset, we considered two probable causes for delayed diagnosis― a delay in the use of DW-MRI, and comorbid ocular disease and onset of visual disturbance.

First, to understand the delayed use of DW-MRI causing a delayed diagnosis, we reviewed 14 patients with pathologically-confirmed MM2C-type sCJD including MM2C±1-, or MM2C+T-type (Table 1) [13–21]. Combined MM2T±C- or MM1 + 2C-type sCJD cases were excluded from our review. The age of onset [range] was 62.3 ± 11.5 [42–83] years, and the disease duration [range] was 20.6 ± 12.8 [5–44] months, which is similar to those previously reported [1]. Duration [range] between onset and diagnosis in these reviewed patients was 9.9 ± 7.8 [1–24] months. Eight out of the 14 patients were diagnosed with MM2C-type sCJD, over 6 months after the onset of symptoms, whereas one patient had remained undiagnosed until death. In the clinically undiagnosed patient, DW-MRI had not been performed. To date, cortical hyperintensity on DW-MRI suggests a clinical diagnosis of CJD, although this finding has not been included in the revised WHO criteria for the diagnosis of CJD. However, cortical or striatum hyperintensity on 1.5-Tesla DW-MRI has been reported to be a reliable diagnostic marker for sCJD [22]. Cortical hyperintensity on DW-MRI was detected in all the reviewed MM2C-type sCJD patients except one where DW-MRI was not performed (13/13, 100%). However, the duration between initial DW-MRI and symptom onset was 9.4 ± 8.1 [1–24] months, and that between the detection of cortical hyperintensity on DW-MRI and diagnosis was 0.5 ± 1.7 [0–6] months. Two of the 14 patients were initially diagnosed with AD or AD-like dementia owing to the slow progressive dementia until the initial DW-MRI was performed. Kransnianski, *et al*. [3] pointed out that some cases of MM2C-type sCJD may be diagnosed as rapidly progressive AD. We considered the duration between symptom onset and initial DW-MRI should be performed to promptly diagnose suspected patients with MM2C-type sCJD.

**Table 1.** Pathologically confirmed MM2 cortical-type sporadic Creutzfeldt-Jakob disease and ocular disease.

Case number	Author	Pathological type of CJD	Age of onset (y)	Age of death (y)	Sex	Disease duration (mo.)	Clinical diagnosis for prion disease after the onset (mo.)	Initial symptoms	Cortical hyperintensities in the initial DW-MRI	Initial DW-MRI perfoemd after the onset (mo.)	Duration between the diagnosis and the initial DW-MRI (mo.)	Ocular disease accompaniment	Initial diagnosis or diagnosis before prion disease
1	Hamaguchi^13)^	MM2C	65	Alive (diagnosis confirmed by brain biopsy)	F	>13	7	Dementia	+	7	0	N.D.	CJD
2	Hamaguchi^13)^	MM2C	75	Alive (diagnosis confirmed by brain biopsy)	F	>28	16	Dementia	+	16	0	N.D.	CJD
3	Hamaguchi^13)^	MM2C+T	65	N.D.	M	14	4	Fall	+	4	0	N.D	CJD
4	Nozaki^14)^	MM2C	65	N.D.	F	20	7	Blurred vision	+	7	0	N.D.	CJD
5	Niimi^15)^, Akagi^16)^	MM2C	67	68	M	5	2	Dementia	+	2	0	N.D.	CJD
6	Saito^17)^	MM2C+T	59	62	M	32	24	Visual disturbance, depression	+	24	0	N.D	Depression, SCD
7	Fernández-Vega^18)^	MM2C+T	N.D.	80	M	27	24	Dementia	+	24	0	N.D.	AD-like dementia
8	Sherstha^19)^	MM2C	42	N.D.	F	30	9	Dementia	+	3	6	N.D.	autoimmune encephalopathy suspected
9	Akagi^16)^	MM2C	68	68	M	5	5	Dementia	+	5	0	N.D	CJD
10	Baiardi^20)^	MM2C+1	70	N.D.	F	26	N.D.	Visual disturbance, campimetric deficit	N.E.	N.E.	N.E.	N.D.	N.D.
11	Baiardi^20)^	MM2C+1	54	N.D.	M	44	12	Visual hallucinations, environmental agnosia, dyscalulia	+	12	0	N.D.	CJD
12	Baiardi^20)^	MM2C	48	N.D.	M	6	2.5	Visual disturbance	+	2.5	0	N.D.	CJD
10	Attaripour Isfahani^21)^	MM2C	49	N.D.	M	8	1	Confusion, dementia	+	1	0	None	CJD
14	Present patient	MM2C	83	85	M	30	15	Visual disturbance	+	15	0	Graucoma and age-related macular degeneration	AD
	AVG ± SD [range]		62.3 ± 11.5 [42–83]			20.6 ± 12.8 [5–44]	9.9 ± 7.8 [1–24]			9.4 ± 8.1 [1–24]	0.5 ± 1.7 [0–6]		

MM2C: MM2 cortical-type of sporadic Creutzfeldt-Jakob disease, MM2C+T: MM2 cortical + thalamic-type sporadic Creutzfelldt-Jakob disease; MM2C+1: MM2 cortical (predominantly) + 1-type sporadic Creutzfeldt-Jakob disease; N.D.; not described; Creutzfeldt-Jakob disease;

DWI: diffusion-weighted MR imge, AD: Alzheimer’s disease; SCD: spinocerebellar degeneration; y: year-old; mo. Months; F: female; M: male; N.E.: not examined; AVG: average; SD: standard deviation.

Second, we consider the accompaniment of ocular disease and the onset of visual disturbance in our patient as additional factors hindering the early diagnosis of MM2C-type sCJD. Visual disturbance associated with sCJD may be present in about 10–15% of patients at onset, and in about 50% of the patients during the course of the disease [23,24]. Especially, the clinical course of isolated visual symptoms without cognitive decline, lasting several weeks or months, is known as the Heidenhain variant form of CJD. According to Table 1, six of the 14 patients were visual symptom onset, and 7 patients were dementia onset of MM2C-type sCJD. The average month of diagnosis after the onset of visual symptom versus the dementia onset was 14.4 ± 9.3 months versus 9.1 ± 8.2 months. The diagnosis of visual symptom onset in patients tends to be more delayed.

Additionally, elderly patients frequently presented with comorbid ocular diseases such as cataract, age-related macular degeneration, or glaucoma. Therefore, the differential diagnosis of CJD from ocular disease is very important, and the accompaniment of ocular disease is one of the causes leading to delay in the accurate diagnosis of CJD.

Based on the clinical course of the disease, the present patient was also diagnosed with posterior cortical atrophy (PCA). Initial symptoms of isolated cortical visual disturbance started in the early-phase of CJD, and subsequently, progresses to dementia. PCA is a syndrome of early visual dysfunction involving the neurodegeneration of the posterior cortex, and its progression ultimately leads to diffuse cognitive dysfunction. Pathologically, PCA is associated to AD, Lewy body disease (DLB), CBD, or prion disease [25]. T1-weighted MRI showed cortical atrophy involving the occipital lobes, and DW-MRI and eZIS study of ^99m^Tc-ECD SPECT showed bilateral hyperintensity and decreased rCBF in the right occipital cortices, respectively. This combination of neuroradiological findings supported a clinical diagnosis of PCA-prion disease. Postmortem analysis revealed the cause of cortical blindness in our patient as MM2C-type sCJD. PCA-AD, PCA-DLB, or PCA-CBD was excluded pathologically. We conclude that the use of DW-MRI is crucial in clinical diagnosing PCA-prion disease.

To date, despite several clinical trials, no effective treatment has been discovered for human prion disease. The rarity, rapidity, and clinical heterogeneity of prion disease affects study enrollment and the ability to measure treatment outcomes [26]. Since MM2C-type sCJD is a slow progressing prion disease [1], we believe this phenotype may become a potential target of clinical trials for devising treatments for prion disease. This phenotype may become a relevant target of clinical trials if the complications pertaining to its delayed diagnosis are overcome.

In conclusion, for early diagnosis of MM2C-type sCJD, clinicians should perform DW-MRI for patients with dementia or cortical visual disturbances.
